# Comparative Learning Curves of Microscope Versus Exoscope: A Preclinical Randomized Crossover Noninferiority Study

**DOI:** 10.3389/fsurg.2022.920252

**Published:** 2022-06-06

**Authors:** Hugo Layard Horsfall, Zeqian Mao, Chan Hee Koh, Danyal Z. Khan, William Muirhead, Danail Stoyanov, Hani J. Marcus

**Affiliations:** ^1^ Victor Horsley Department of Neurosurgery, National Hospital for Neurology and Neurosurgery, London, United Kingdom; ^2^ Wellcome/EPSRC Centre for Interventional and Surgical Sciences, University College London, London, United Kingdom

**Keywords:** microscope, exoscope, neurosurgery, learning curve, innovation, education, surgery

## Abstract

**Background:**

An exoscope heralds a new era of optics in surgery. However, there is limited quantitative evidence describing and comparing the learning curve.

**Objectives:**

This study aimed to investigate the learning curve, plateau, and rate of novice surgeons using an Olympus ORBEYE exoscope compared to an operating microscope (Carl Zeiss OPMI PENTERO or KINEVO 900).

**Methods:**

A preclinical, randomized, crossover, noninferiority trial assessed the performance of seventeen novice and seven expert surgeons completing the microsurgical grape dissection task “Star’s the limit.” A standardized star was drawn on a grape using a stencil with a 5 mm edge length. Participants cut the star and peeled the star-shaped skin off the grape with microscissors and forceps while minimizing damage to the grape flesh. Participants repeated the task 20 times consecutively for each optical device. Learning was assessed using model functions such as the Weibull function, and the cognitive workload was assessed with the NASA Task Load Index (NASA-TLX).

**Results:**

Seventeen novice (male:female 12:5; median years of training 0.4 [0–2.8 years]) and six expert (male:female 4:2; median years of training 10 [8.9–24 years]) surgeons were recruited. “Star’s the limit” was validated using a performance score that gave a threshold of expert performance of 70 (0–100). The learning rate (ORBEYE −0.94 ± 0.37; microscope −1.30 ± 0.46) and learning plateau (ORBEYE 64.89 ± 8.81; microscope 65.93 ± 9.44) of the ORBEYE were significantly noninferior compared to those of the microscope group (*p* = 0.009; *p* = 0.027, respectively). The cognitive workload on NASA-TLX was higher for the ORBEYE. Novices preferred the freedom of movement and ergonomics of the ORBEYE but preferred the visualization of the microscope.

**Conclusions:**

This is the first study to quantify the ORBEYE learning curve and the first randomized controlled trial to compare the ORBEYE learning curve to that of the microscope. The plateau performance and learning rate of the ORBEYE are significantly noninferior to those of the microscope in a preclinical grape dissection task. This study also supports the ergonomics of the ORBEYE as reported in preliminary observational studies and highlights visualization as a focus for further development.

## Introduction

The operating microscope pioneered in the 1950s by Yasagil (1) remains the gold standard for microneurosurgery. More recently, an “exoscope” system has been introduced as a potential alternative to the microscope (2). Suggested benefits of an exoscope include improved ergonomics and being a valuable educational tool (2–5). A newly developed exoscope is an ORBEYE (Olympus, Tokyo, Japan, 2017), equipped with 3D optics, 4 K imaging quality, and comparable field of view and depth of field to those of the microscope.

The safe introduction of novel technology into clinical practice is central to reducing patient harm (6, 7). Surgeons gain procedural competence as their experience increases with a device (8, 9). This relationship between learning effort and the outcome can be represented using learning curves (10, 11). Factors that affect the learning curve are the initial skill level, the learning rate, and the final skill level achieved—known as the learning plateau (10, 12, 13). Understanding learning curves, both at individual and system levels, is crucial for assessing a new surgical technique or technology, informing surgical training, and evaluating procedures in practice (14, 15). Previous comparative studies suggest the presence of a learning curve for experienced surgeons with the ORBEYE, but there has been no attempt at quantification of the learning curve nor a direct comparison of the learning curve for both the microscope and ORBEYE in relation to novice surgeons (5, 16–18).

We explored the learning rate, learning plateau, and cognitive load of novice surgeons performing a validated microsurgical grape dissection task.

We performed a microsurgical grape dissection task to explore the learning rate, learning plateau, and cognitive load of novice surgeons with limited experience of both the microscope and OREYE; the learning curve of the ORBEYE is not inferior to that of the traditional microscope.

## Methods

### Protocol and Ethics

The protocol was registered with the local Clinical Governance Committee and was approved by the Institutional Review Board. The Consolidated Standards of Reporting Trials Statement (19) (CONSORT) with noninferiority extension was used.

### Participants

Novice and expert surgeons were recruited from a university hospital. Novice surgeons had not performed any operative cases on either the microscope or the ORBEYE. Expert surgeons had completed their neurosurgical training (20, 21). Informed written consent was obtained.

### Sample Size

A target sample size of 12 novices was set. Owing to pragmatic constraints and the lack of applicable pilot data, no power calculation was undertaken, but such a number was deemed appropriate based on previous similar studies (20–23).

### Randomization

Novice surgeons were randomly allocated to start on either optical device before crossing over. Permuted blocked randomization (block size 2 and 4) using a computer-generated sequence. One author (ZM) performed sequence generation and implementation. Blinding was not possible due to the nature of optical devices.

### Interventions

#### Microsurgical Grape Dissection Task: “Star’s the Limit”

**Figure 1:**
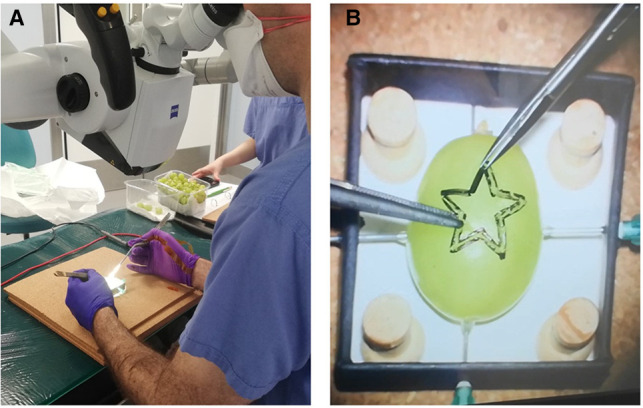
Microsurgical grape dissection task “Star’s the limit” setup. (**A**) Microscope trial. (**B**) “Star’s the limit.” Note the grape has a homogeneous shape, drawn on by stencil, and secured using needles to ensure constant position across the trials.

Participants performed a validated microsurgical task “Star’s the limit” (24). A standardized star is drawn on a grape using a stencil with a 5 mm edge length. Participants cut the star and peeled the star-shaped skin off the grape with microscissors and forceps while minimizing damage to the grape flesh (Figure 1). Each novice repeated the task 20 times consecutively before changing the device and repeating the task a further 20 times consecutively. The microsurgical task was validated by experts who repeated the task 20 times consecutively using the microscope only. No feedback or teaching was provided to the participants during the task. If participants were not able to finish within 5 min, they were told to stop, and the next repetition would begin. The microscopes used were an OPMI PENTERO or a KINEVO 900 (Carl Zeiss Co, Oberkochen, Germany).

### Outcomes

#### Primary Outcomes: Learning Plateau, Learning Rate, and Learning Curves

The performance of participants in the task was assessed using a five-item rubric: (1) time to completion; (2) completeness of dissection; (3) degree of grape flesh attached to the star; (4) number of edges incised within the drawn lines; and (5) perforation of grape flesh with the instruments (Table 1). The same assessor (ZM) prepared all 820 grapes and assessed all 17 novice and 7 expert surgeons to reduce scoring intravariability. The raw scores were combined into a composite performance score for each repetition. There was a modification to the performance score algorithm from the original protocol (OpenEd@UCL repository; https://open-education-repository.ucl.ac.uk//620/). The performance score was calculated as follows:

Performancescore=90–0.3×(Timetaken)+0.5×Edgescore

**Table 1:** Microsurgical grape dissection task “Star’s the limit” grading rubric.

Items	Descriptions
Time to Complete	The time to completion (seconds) is recorded, up to 5 min for each repetition; otherwise, participants are told to stop
Completeness of the Dissected Star	Defined as star-shaped grape skin is obtained
	0 for failure
	1 for success
Clean Star with No Flesh – “flesh score”	The dissected star needs to be “clean skin” without flesh attached
	0 points for a lot of flesh or no star obtained
	1 point for some flesh
	2 points for no flesh
Edge within Limit – “edge score”	Incision needs to be made within the drawn line
	Both the dissected star and the remaining grape is examined; 1 point for the existence of the blackish on each edge
	If no star obtained, up to 10 points since only the main grape can be assessed
	If star obtained, up to 20 points
Perforation	The number of perforations made is recorded
	1 point deduction for every perforation into the deep grape flesh

Numerous different learning curve models were tested (Appendix 1). The best-fitting curve was selected using log-likelihood. All curves tested are characterized by the rate of improvement decreasing over time (Appendix 1). The curves were fitted with nonlinear mixed-effects models. Fixed effects were used to

1. investigate the difference between the microscope and ORBEYE and
2. establish the coefficients of the curves.

Random effects were used to account for

1. nonindependence of the data from the same subject and
2. random variations in coefficients between subjects.

The fixed effects of the model output gave the curves averaged across each group. These fixed-effects outputs were used to test the noninferiority of the asymptote and the learning rates of the ORBEYE group compared to thosed of the microscope group. The inferiority threshold was set *a priori* at 20%, and a one-tailed *t*-test was used against this inferiority margin to give the plateau performance.

Finally, crossover analysis was performed to evaluate whether the starting optical device (ORBEYE or microscope) had an impact on novices’ performance. The final five trials before and after crossover were considered. The performance score was investigated before and after crossover (fixed-effects), and the difference between each group (fixed-effects) was taken into account by the subject (random-effects). The analysis was performed using linear mixed-effects regression.

#### Secondary Outcomes: Subjective Impression of Optical Devices

The perceived workload was assessed using the NASA Raw Task Load Index (NASA R-TLX) (25, 26) (Appendix 2). Within the NASA R-TLX are six domains: mental, physical, and temporal demands, performance, effort, and frustration, and these are rated using a 20-point scale. Participants completed the NASA R-TLX immediately after finishing the task. The domain score and total score were used for the secondary outcome. Novice surgeons also reported their subjective impression of the microscope and ORBEYE (Appendix 3).

### Statistical Methods

Curve fitting was performed using R *v4.1.2* (27); linear mixed-effects regression analysis was made using packages lme4 v1.1 (28), lmerTest v3.1 (29), and nlme v3.1 (30). Noninferiority testing of the learning curves was conducted using the outputs for the estimates, standard errors, and the degrees of freedom of the growth curve coefficients by utilizing the base R functions for Student’s *t* distributions. Subjective impression analysis was conducted using JASP v0.14.1 and GraphPad Prism v9.2.0. Data are expressed as mean ± 95% confidence intervals or median ± IQR. The threshold for statistical significance was set at *α* < 0.05. Adjustments for multiple comparisons were made using the Benjamini–Hochberg method for false discovery rates.

## Results

### Participants

Seventeen novice and seven expert surgeons were recruited (Appendix 4). The novice surgeons (male:female 12:5) had completed a median of 0.4 years of training (0–2.8 years). No novice surgeon had not performed any microsurgical cases using either the ORBEYE or a microscope. The expert surgeons (male:female 4:2) had a median 10 years of training (8.9–24 years) and completed the “Star’s the limit” task using the microscope only to validate the grape dissection model.

### Validation of the Composite Performance Score

The time to task completion was the slowest during the first attempts and plateaued by the 16th attempt (Appendix 5). To compare “absolute novice” and “absolute expert” for validation purposes, the first five attempts for novices and the final five attempts for experts were considered. The time taken for task completion by novices and experts demonstrates that participant 2 (expert) and 10 (novice) are outliers and was excluded from the validation analysis.

The Akaike information criterion (AIC) was used to select the best predictive model for discrimination between novice and expert (Table 2). The best performing model was time taken to task completion alone (AIC: 13.1) and the second-best performing model was time taken + edge score (AIC: 15.0). We elected to utilize the latter model despite the higher AIC value to ensure penalization for a fast performance if performed poorly. A validated performance score was created with a threshold of expert performance of 70 (0–100) (Appendix 6).

**Table 2:** Summary of best selective model function for discrimination between novice and expert performances.

	AIC	logLik
(1−Total time/300)	13.14999	−3.574996
(1−Total time/300)+Edge	15.01496	−3.507481
(1−Total time/300)+Accuracy score	15.05006	−3.525028
(1−Total time/300)+Clean star	15.14999	−3.574994
(1−Total time/300)+Clean star + Edge	16.79633	−3.398165
(1−Total time/300)+Edge + Perforations	17.01496	−3.507480

*AIC, Akaike information criterion; logLik, log likelihood ratio*.

### Investigating the Learning Curve

**Figure 2:**
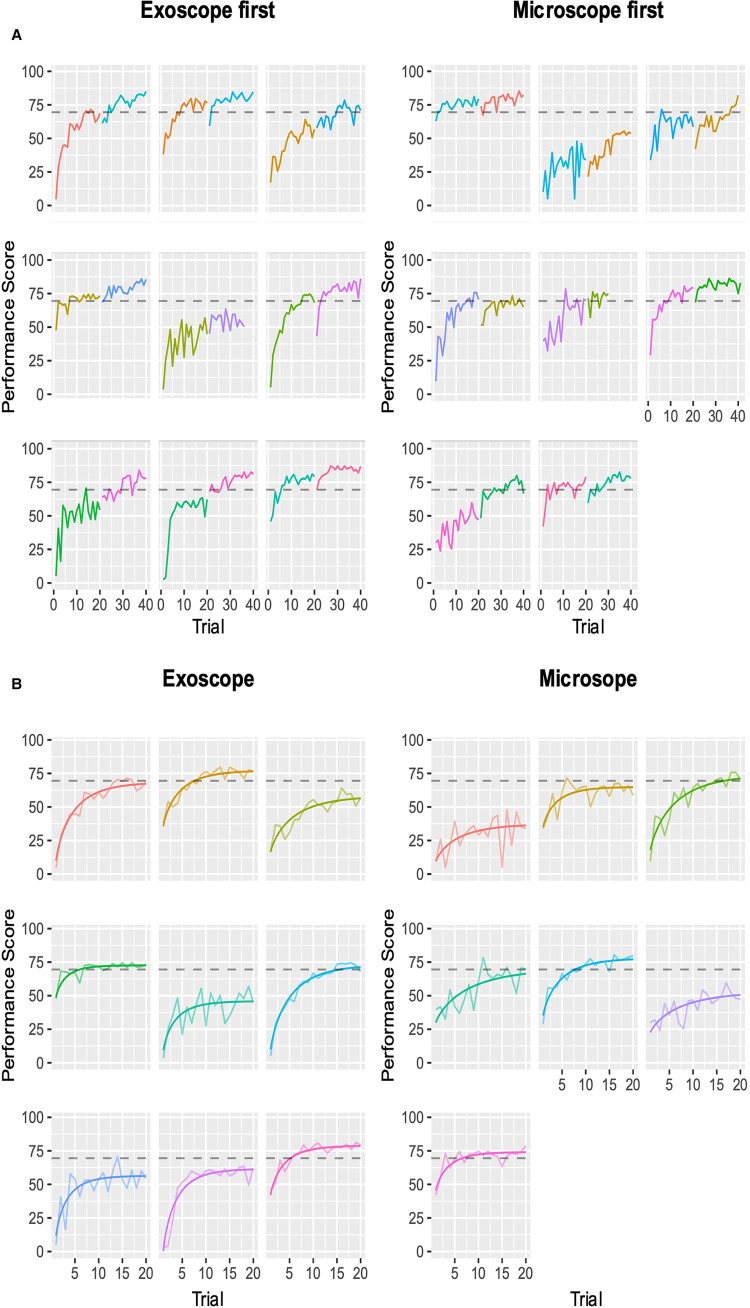
Composite performance score of novice surgeons completing the microsurgical grape dissection task with a threshold for expert performance (gray dashed line 70; 0–100). Each graph represents a separate novice. In total, participants completed 20 repetitions of the task on each device consecutively. (**A**) Novice surgeons’ performance scores plotted against the number of trials performed, with the group starting with the ORBEYE and microscope. The first colored graph represents the first device, and the second colored graph represents the crossover to the second device. (**B**) Modeled learning curves using the modified Weibull function.

**Figure 3:**
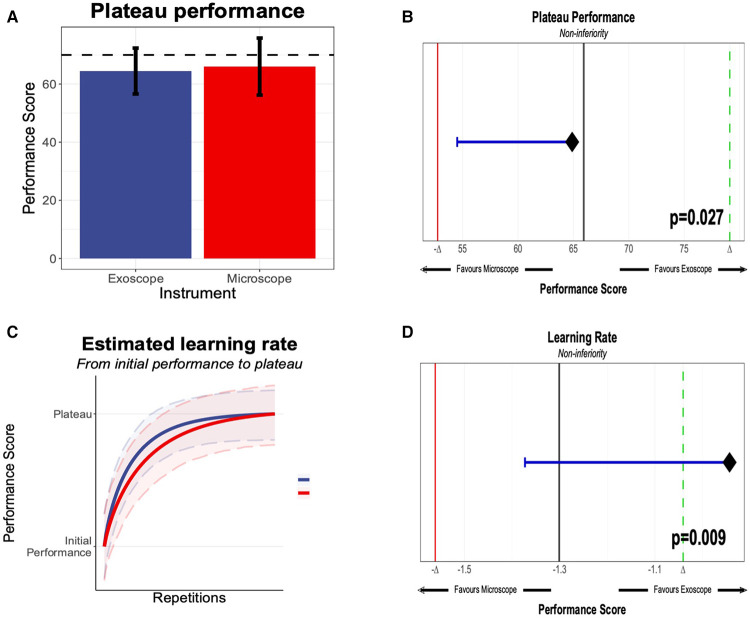
(**A**) Plateau performance between the novices, starting the “Star’s the limit” task on the exoscope or the microscope. The performance score was 0–100, generated through methods outlined in section Methods, with an expert threshold score of 70. (**B**) Significant noninferiority of the microscope and exoscope based on novice performance compared against the expert performance. (**C**) Learning rate of novices on the exoscope or the microscope. (**D**) Learning rate and statistical noninferiority of the exoscope compared to the microscope.

We evaluated the seven different models, and the best fit was the modified Weibull function (AIC 2389.013; log-likelihood −1181.506) (Figure 2; Appendix 7). The primary outcome of the learning rate (ORBEYE −0.94 ± 0.37; microscope −1.30 ± 0.46) and learning plateau (ORBEYE 64.89 ± 8.81; microscope 65.93 ± 9.44) of the ORBEYE was significantly noninferior compared to that of the microscope group (*p* = 0.009; *p* = 0.027, respectively) (Figure 3). If considering participants crossing over to the ORBEYE from the microscope, the plateau is not (but nearly significant) noninferior (*p* = 0.055). ANOVA analysis demonstrates that performance significantly improved after crossover in both groups (microscope to ORBEYE and ORBEYE to microscope).

### Workload Assessments

**Figure 4:**
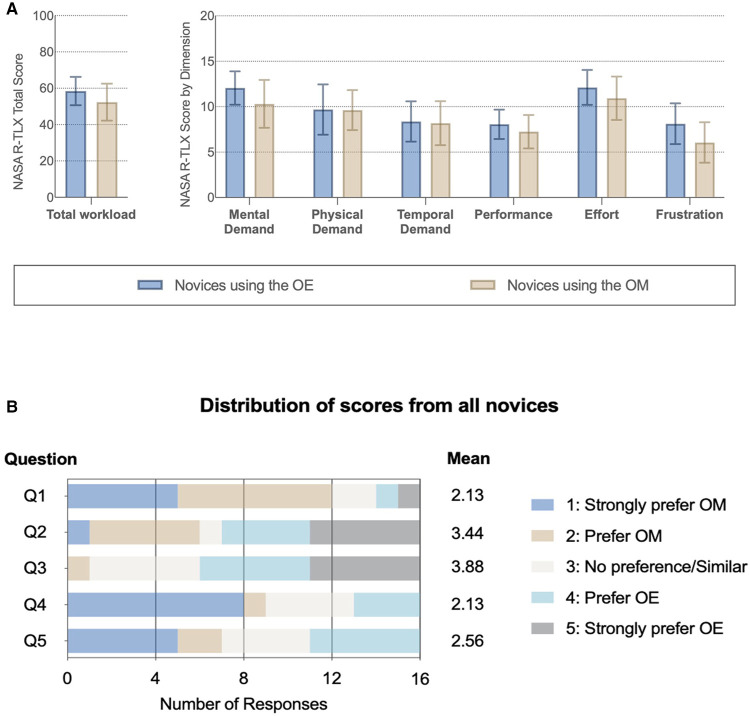
Subjective impression of the optical device. (**A**) NASA Raw Task Load Index (NASA R-TLX) score for each dimension and total workload compared between novices with different instruments. (**B**) Subjective questionnaire (Supplementary Material Table 2): Q1: better visualization; Q2: greater freedom of movement; Q3 more comfortable; Q4: easier to perform a task with; and Q5: prefer to use in the future.

The NASA R-TLX demonstrates that mental demand, performance, effort, and frustration scores were not statistically different between the ORBEYE and the microscope (Figure 4A). Analysis of variance (repeated-measure two-way ANOVA) showed no significant main effect of device and group assignment on the workload scores, and a significant interaction was only found in the temporal demand score (*p* < .05). However, post-hoc comparisons with FDR adjustments did not find significant results.

### Subjective Impression

Novice surgeons answered the subjective questionnaire following task completion on each device (Figure 4B; Appendix 8). For Q1, 75% (12/16) of novices strongly preferred or preferred the microscope compared to the ORBEYE regarding visualization. Further, 55% (9/16) strongly preferred or preferred the microscope compared to the ORBEYE for task completion. Novices strongly preferred or preferred the greater movement (55%; 9/16) and comfortable ergonomics (63%; 10/16) of the ORBEYE compared to the microscope. Seven novices compared to five novices would prefer to use the microscope rather than the ORBEYE in the future (Figure 4B).

## Discussion

### Interpretation

This preclinical, randomized, crossover noninferiority trial provides robust data that quantifies for the first time that the learning curve of the ORBEYE is statistically noninferior regarding the learning rate and learning plateau compared to that of the traditional operating microscope in novice surgeons (Figure 3).

The modified Weibull function was the best fit to model learning curves using the composite performance scores. The microsurgical task was satisfactory at discriminating between novice and expert surgeons using the composite performance score. This adds validity to the modeled learning curves as representatives of real-world learning curves.

There was no significant difference in the cognitive workload of the optical devices using the NASA R-TLX. The total workload score was 61 (IQR: 44.75–72.75) for the ORBEYEE and 53 (IQR: 38.75–63) for the microscope. Analysis of variance demonstrated no significant difference in the scores depending on the optical device the novice surgeon started on.

Subjective assessment of the optic devices found that novices preferred the visualization, ease of task completion, and preferential future use of the microscope, while they preferred the ergonomics and greater freedom of movement of the ORBEYE.

Taken together, and considering the microscope has been standard practice for decades, this study does not intend to definitively state that the ORBEYE should replace the microscope. Instead, the ORBEYE has a similar learning plateau and learning rate to the microscope in novice surgeons on a preclinical task; further work must be undertaken to facilitate safe, comparative clinical studies.

### Comparison with the Literature

The “learning curve” is frequently used in surgical education literature and represents the relationship between learning effort and the outcome (11, 12, 15, 31). Understanding the learning curve, rate, and plateau provides a mechanism for understanding the development of procedural competency (7, 15). The learning curve is vital when introducing novel technology to surgical practice and should be established before any definitive comparative clinical trials (6, 32, 33). The current ORBEYE literature describes the learning curve subjectively or is inferred from a sample of experienced surgeons (16, 17, 34, 35). The present study provides quantitative data that models the learning curve for novices for both the microscope and the ORBEYE. We demonstrate no significant inferiority for either optical device. This should encourage the international community to ensure trainees develop skills for both optical devices.

The subjective impression from the novice surgeons in this study prefers or strongly prefers the ORBEYE’s freedom of movement and comfortable position. This supports existing literature and descriptive studies (35, 35–37). Regarding visualization, participants strongly preferred or preferred the microscope compared to the ORBEYE (Figure 4). Microscope preference might have been influenced by obstruction of the line of sight or increased “noise” in peripheral vision while completing novel tasks. The subjective feedback supports future ORBEYE development to enhance their visualization. Future advances to the ORBEYE may add further functionality, including the possibility of augmenting data flow due to the digital nature of the ORBEYE, permitting interoperability with other technological innovations such as augmented reality or computer vision.

### Strengths and Limitations

The aim was to compare the learning curves of the ORBEYE and microscope in novices. Previous studies ask experts to perform simulations using the ORBEYE or microscope or ask for subjective feedback after performing surgery with the exoscope. This introduces bias based on their previous experience and likely established preference. It was therefore considered more robust to use a single, large, homogeneous group of novice surgeons with limited surgical experience, rather than a small group of experts with varying experience of optical device and technical expertise. We did validate the task with expert surgeons to characterize the learning curve for novices. To ensure we avoided any learning curve with the visualization device, we felt it best for expert surgeons to complete the task on the device they were most familiar with, in this case, the microscope. The validated low-fidelity task was also appropriate to characterize the learning curve, as it was not too easy that novices could perform perfectly but not too challenging that a plateau was not achieved at the end of 20 consecutive repetitions. Our methods were also published *a priori*; participants were randomized to reduce the risk of bias and modeled over 60 variants of the performance score.

A limitation was using a low-fidelity microsurgical grape dissection task. Although this has precedence within the literature, our findings require further validation with higher fidelity models when evaluating an expert’s learning curve, such as suturing or anastomoses. The novice sample size is small, although again concordant with the literature. Finally, no participant did not crossover; therefore, we cannot control for the effect of switching optical devices.

## Conclusion

This is the first study to quantify the ORBEYE learning curve and the first randomized controlled trial to compare the ORBEYE learning curve to the microscope. The plateau performance and learning rate of the ORBEYE are significantly noninferior to those of the microscope in a preclinical grape dissection task. This study also supports the ergonomics of the ORBEYE as reported in preliminary observational studies and highlights visualization as a focus for further future development.

## Data Availability

The raw data supporting the conclusions of this article/Supplementary Material will be made available by the authors without undue reservation.
