# Supplementary material for: Comparative Learning Curves of Microscope Versus Exoscope: A Preclinical Randomized Crossover Noninferiority Study
Source: Front Surg. 2022 Jun 6;9:920252. doi: 10.3389/fsurg.2022.920252 (PMC9316615; doi:10.3389/fsurg.2022.920252)
Supplement: Supplementary file 1 [file Table_1_v1.docx]

Appendix

**1.** Summary of function tested to model the learning curve.

**
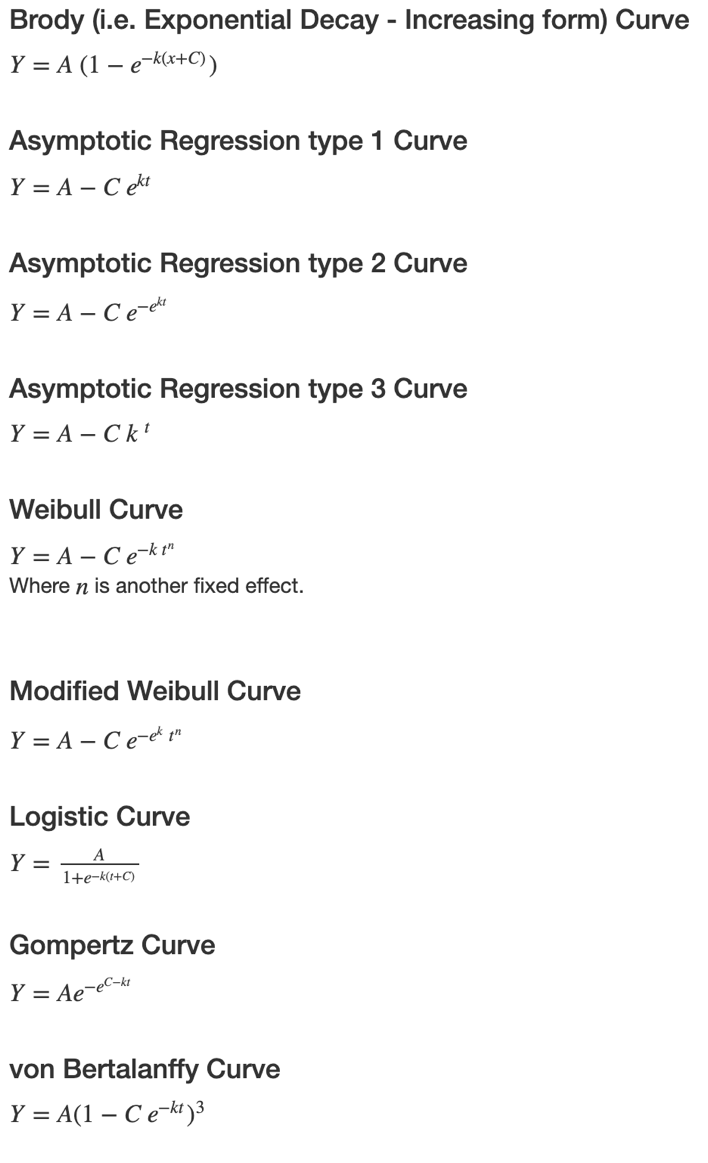
**

The curves utilized either all or some combination of the following parameters:

- Y: performance score
- t: time (i.e. trial/repetitions/attempts)
- A: asymptote (i.e. Plateau)
- k: learning rate
- C: constant (where needed)

**Appendix 2.** NASA R-TLX

| **Task Load Index** | | | | | | | | | | | | | | |
| --- | --- | --- | --- | --- | --- | --- | --- | --- | --- | --- | --- | --- | --- | --- |
| Date *(complete by observer)*: | | | | | | | Subject ID *(complete by observer)*: | | | | | | | |
| Instrument (circle): **Penetro / Kinevo / ORBEYE** / if others, state: | | | | | | | | | | | | | | |
|  | | | | | | | | | | | | | | |
| *Instruction: please evaluate the task by marking ‘‘****X****’’ on each scale at the point which best ﬁts your experience. Please read the scale description and endpoint carefully.* | | | | | | | | | | | | | | |
| **Mental Demand** | | | | | | | | | | | | | | |
| How much mental and perceptual activity was required (e.g., thinking, deciding, calculating, remembering, looking, searching, etc.)? Was the task easy or demanding, simple or complex, exacting or forgiving? | | | | | | | | | | | | | | |
| 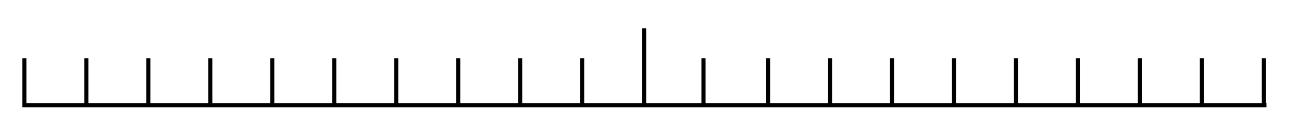 | | | | | | | | | | | | | | |
|  |  |  |  |  |  | Very Low | | Very High |  |  |  |  |  |  |
| **Physical Demand** | | | | | | | | | | | | | | |
| How much physical activity was required (e.g., pushing, pulling, turning, controlling, activating, etc.)? Was the task easy or demanding, slow or brisk, slack or strenuous, restful or laborious? | | | | | | | | | | | | | | |
| 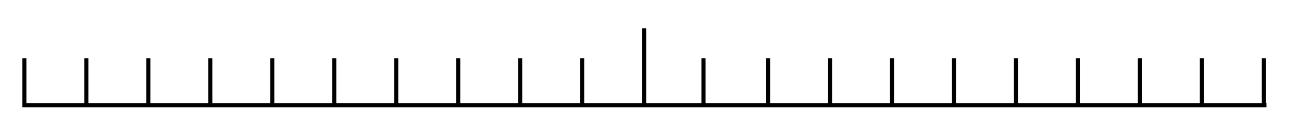 | | | | | | | | | | | | | | |
|  |  |  |  |  |  | Very Low | | Very High |  |  |  |  |  |  |
| **Temporal Demand** | | | | | | | | | | | | | | |
| How much time pressure did you feel due to the rate or pace at which the tasks or task elements occurred? Was the pace slow and leisurely or rapid and frantic? | | | | | | | | | | | | | | |
| 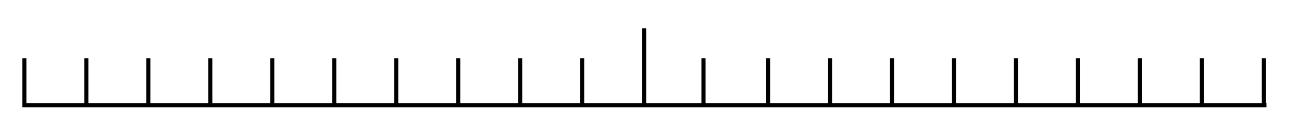 | | | | | | | | | | | | | | |
|  |  |  |  |  |  | Very Low | | Very High |  |  |  |  |  |  |
| **Performance** | | | | | | | | | | | | | | |
| How successful do you think you were in accomplishing the goals of the task set by the experimenter (or yourself)? How satisfied were you with your performance in accomplishing these goals? | | | | | | | | | | | | | | |
| 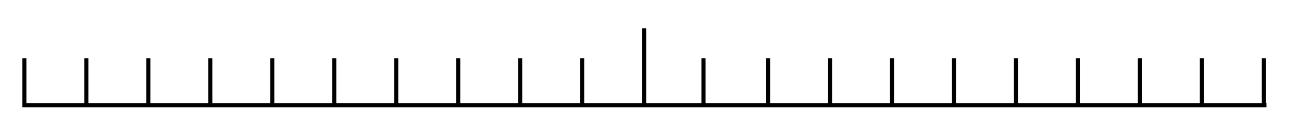 | | | | | | | | | | | | | | |
|  |  |  |  |  |  | Perfect | | Failure |  |  |  |  |  |  |
| **Effort** | | | | | | | | | | | | | | |
| How hard did you have to work (mentally and physically) to accomplish your level of performance? | | | | | | | | | | | | | | |
| 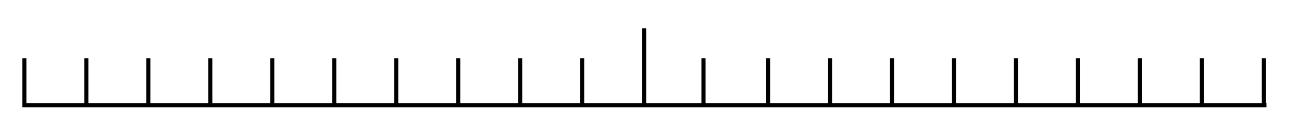 | | | | | | | | | | | | | | |
|  |  |  |  |  |  | Very Low | | Very High |  |  |  |  |  |  |
| **Frustration** | | | | | | | | | | | | | | |
| How insecure, discouraged, irritated, stressed, and annoyed or secure, gratified, content, relaxed, and complacent did you feel during the task? | | | | | | | | | | | | | | |
| 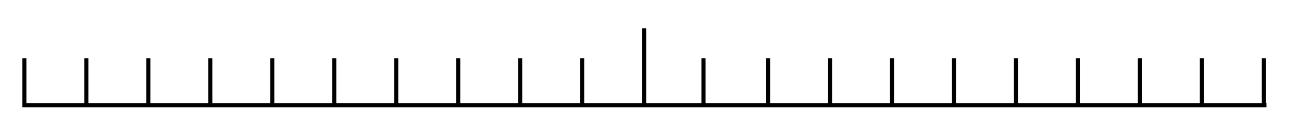 | | | | | | | | | | | | | | |
|  |  |  |  |  |  | Very Low | | Very High |  |  |  |  |  |  |

| Questions Comparing the Subjective Impression of the Operating Microscope and the ORBEYE | |
| --- | --- |
| 1 | Which one provides better visualisation? (Consider image sharpness and brightness) |
| 2 | Which one provides greater freedom of movement? |
| 3 | Which one allows a more comfortable posture? (Consider ergonomic comfort) |
| 4 | Which one felt easier to perform the task with? |
| 5 | Which one would you prefer to use in the future? |
| 6 | Any other comments about the experience with two instruments |

The questionnaire used for evaluation comprised 6 questions. Question 1-5 were based on rating scale (1 for “Strongly prefer OM”, 2 for “Prefer OM”, 3 for “No preference/Similar”, 4 for “Prefer OE”, 5 for “Strongly prefer OE”). Question 6 was descriptive.

**Appendix 3**. Subjective qualitative feedback on optical device from novice surgeons.

**CONSORT 2010 Flow Diagram**

Assessed for eligibility (n=17) NB. 7 expert surgeons also recruited, but not randomised as used to validate performance metric

Allocated to operating microscope then crossover to OrbEye (n=8)

Excluded (n=0)

## Follow-Up

Analysed (n=7)
♦ Excluded from analysis (n=1, outlier)

## Analysis

Analysed (n=9)
♦ Excluded from analysis (n=0)

No follow-up required

No follow-up required

## Enrollment

## Allocation

Allocated to OrbEye then crossover to operating microscope (n=9)

Randomized (n=17)

**Appendix 4.** CONSORT Flow Diagram for trial methodology.

**Appendix 5.** Novice and expert performance at the microsurgical grape dissection task using the microscope. (A) Time taken (s) for both novice (n=8) and expert (n=7) surgeons. (B) The same novice’s and expert’s performance at microsurgical grape dissection task using the microscope plotted against the threshold for expert performance (grey dashed line 70; 0-100)


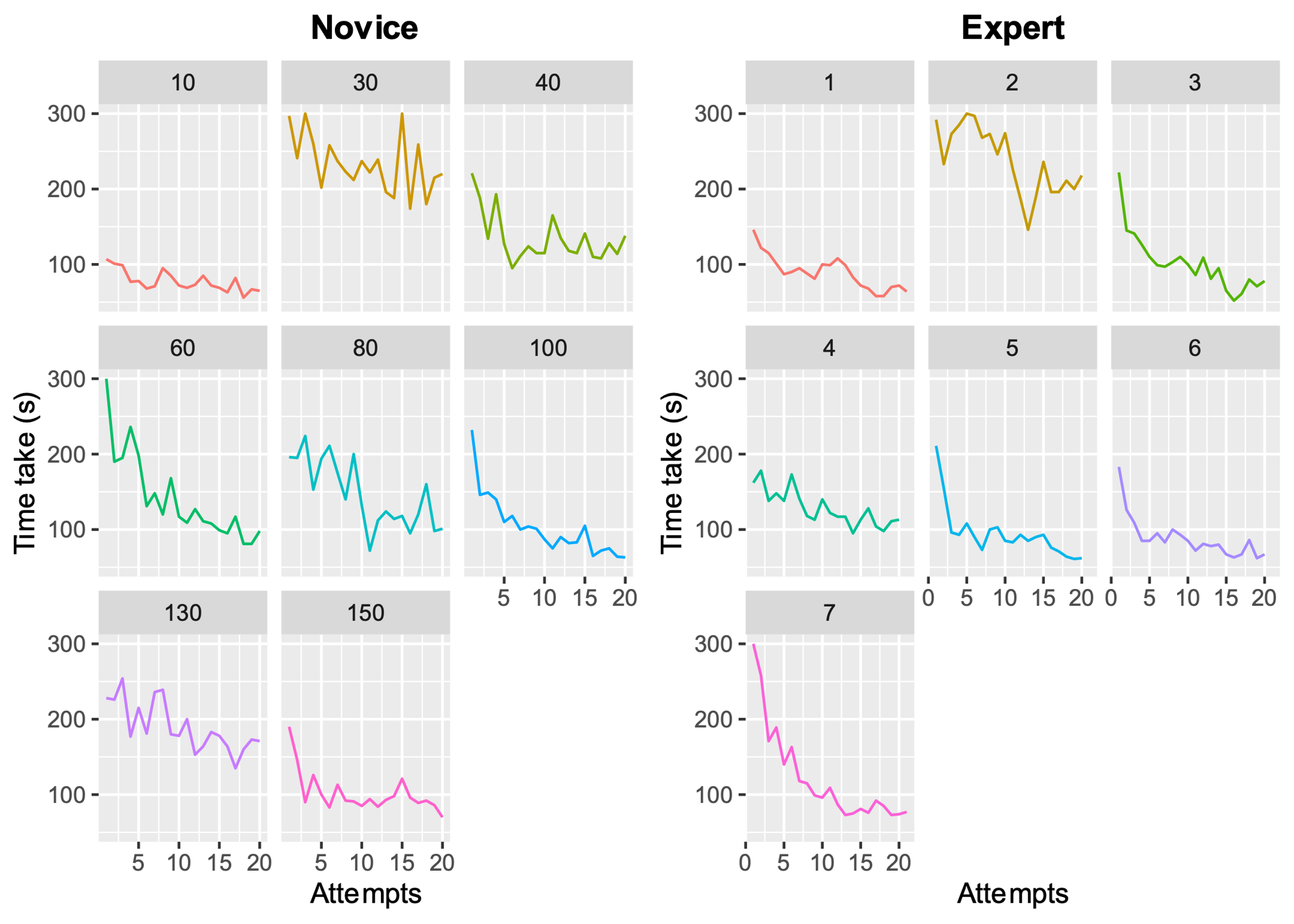

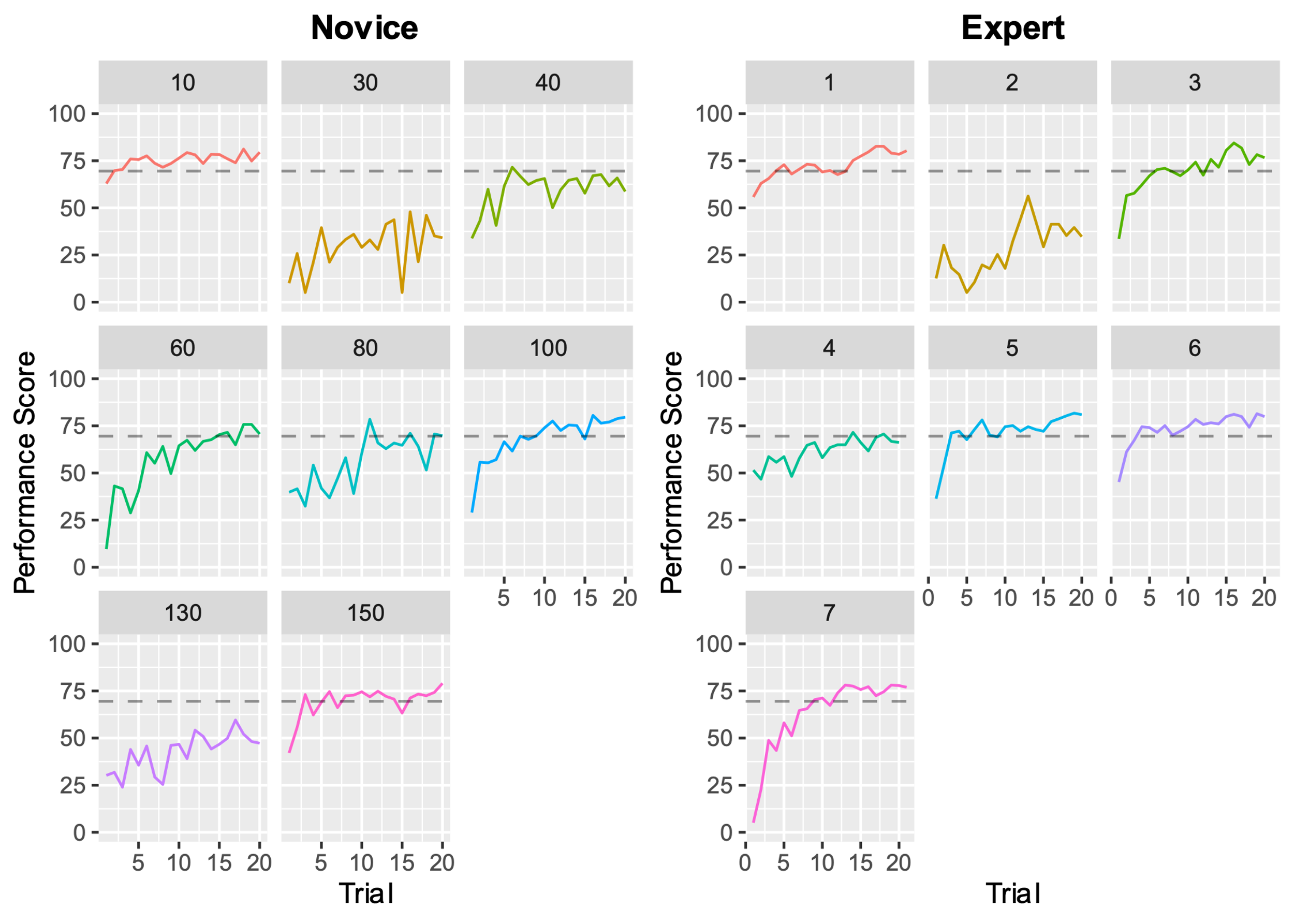

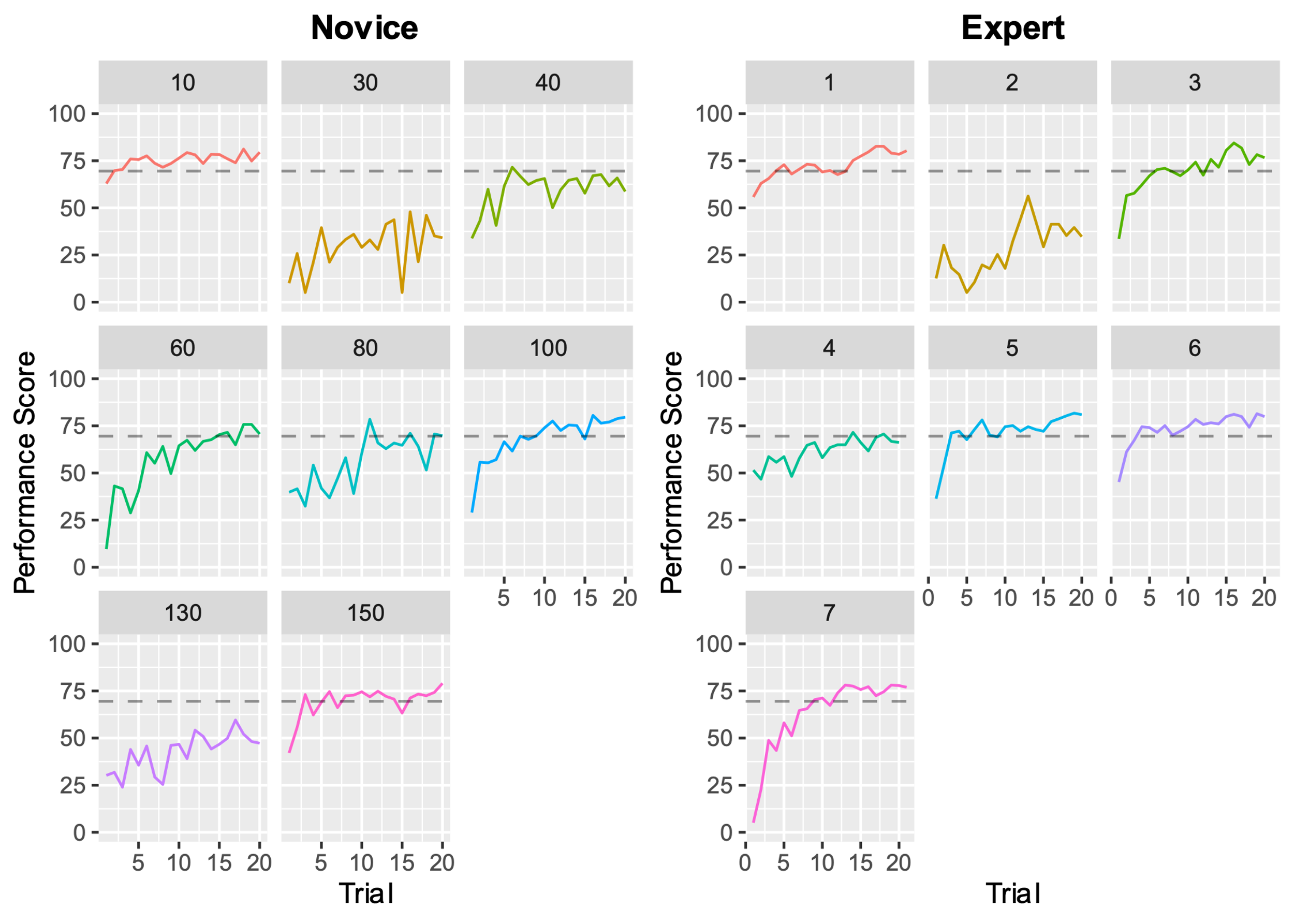


**A**

**B**

**Appendix 6.** Creation of validated performance score and summary of “check score” metric

The raw values produced by the AIC can theoretically range from −∞ to ∞. These values get submitted into sigmoid function, which will produce an output ranging from 0 to 1 that gives the “*probability*” of this performance being by an expert. Inputting 0 into sigmoid function will output 0.5, or the equal likelihood of novice vs expert. Taken together, the raw scores were scaled to make a “real world” score that ranged from 0 – 100. The theoretical maximal performance is *0 seconds to complete task* and maximum *edge score of 20*, giving a raw score of 114.831. The theoretical minimal performance is *300 seconds to complete task* and minimal *edge score of 0*, giving a raw score of -261.747.

Taken together, we added 261.747 (the theoretical minimum performance) to all raw scores to produce a minimum of zero. We scaled all values by the theoretical maximal and minimal performance scores to give a maximum value of 1, and multiplied by 100, resulting in scores that ranged from 0 – 100. A score of 100 x 261.747 / 376.578 = 69.507 would be the threshold of an expert performance, demonstrated by the grey dotted line (Figure 2) comparing novice and expert performance score on the microscope, and comparing the ORBEYE and microscope in novices (Figure 3).

We further explored if time to task completion and edge score can discriminate between “experienced novices” and “experienced experts”, participants that had completed > 15 trials of the grape dissection task, using the “check score”. Using this formula, 98 – 0.3 x *time taken* + 0.1 x *edge score* gave a threshold of expert performance of 64. This score was better at discriminating between “experienced” novices (rather than “absolute” novices) and experts. Using the check score, time taken has a greater relative weighting than the edge score. The coefficient for time taken is 0.3 in both groups, but the edge score is 0.1 compared to 0.5. This means the edge score becomes five times less important when it comes to distinguishing “experienced novices” novices and experts, in comparison to “absolute” novices and experts. Further, when graphically representing the “check score” against the performance score and the plots looked similar.

**Appendix 7**. Modelling the learning curves.

|  | Value | Standard error |  | p value |  |
| --- | --- | --- | --- | --- | --- |
| A | 67.115 | 4.710 |  | 0.000 |  |
| a1 | -1.939 | 6.176 |  | 0.758 |  |
| k | -1.280 | 0.230 |  | 0.000 |  |
| x1 | 0.356 | 0.256 |  | 0.166 |  |
| C | 40.020 | 4.173 |  | 0.000 |  |
| pwr | 0.842 | 0.093 |  | 0.00 |  |

- A is the asymptote of microscope group
- a1 is the difference in asymptote of the exoscope group
- k is the learning rate of the microscope group
- x1 is the difference in learning rate of the exoscope group
- C is a constant such that the initial performance score in the first trial is expected to be A - C
- pwr is the the constant that goes into trial^pwr^ (written above as t^n^)

**Appendix 8.** NASA R-TLX score for each instrument by novices.

|  | Novices when using the OE | Novices when using the OM |
| --- | --- | --- |
| Mental demand | 12.5 (10.25-14.75) | 11.5 (5.5-14.75) |
| Physical demand | 9 (4.25-14.5) | 9 (6-13.75) |
| Temporal demand | 7.5 (6-12) | 8.5 (4.25-12) |
| Performance | 7 (5-10.75) | 6.5 (4.5-9.5) |
| Effort | 13 (10.25-15) | 12 (7.25-14) |
| Frustration | 7 (5.25-12) | 6 (3-8) |
| Total workload | 61 (44.75-72.75) | 53 (38.75-63) |

N = 16. Values are median (IQR). The score by dimension is out of 20. The total score is out of 100.
